# Major dietary patterns and food groups in relation to rheumatoid arthritis in newly diagnosed patients

**DOI:** 10.1002/fsn3.1938

**Published:** 2020-10-12

**Authors:** Negin Mosalmanzadeh, Sajedeh Jandari, Davood Soleimani, Mohammad Reza Shadmand Foumani Moghadam, Fatemeh Khorramrouz, Asie Araste, Seyed Fatemeh Molavi, Reihane fakhlaie, Mohammadhassan Jokar, Reza Rezvani

**Affiliations:** ^1^ Department of Nutrition Sciences Varastegan Institute for Medical Sciences Mashhad Iran; ^2^ Department of Nutrition Faculty of Medicine Mashhad University of Medical Sciences Mashhad Iran; ^3^ Department of Nutritional Sciences School of Nutrition Sciences and Food Technology Kermanshah University of Medical Sciences Kermanshah Iran; ^4^ Rheumatic Diseases Research Center School of Medicine Mashhad University of Medical Sciences Mashhad Iran

**Keywords:** healthy dietary pattern, rheumatoid arthritis, traditional dietary pattern, Western dietary pattern

## Abstract

**Background:**

Evidence suggests that dietary patterns might act as environmental triggers in the development of chronic disorders such as rheumatoid arthritis (RA). However, data regarding the relationship between food patterns and RA are still limited and conflicting. In the current study, the authors aim to evaluate a link between major dietary patterns and RA in new case patients.

**Methods:**

This study was conducted in a case–control manner on 50 patients with newly diagnosed RA and 100 healthy individuals living in Mashhad, Iran. The individuals’ dietary intake was assessed using a validated food frequency questionnaire (FFQ). The major dietary patterns were identified using factor analysis based on data from FFQ. Multivariable‐adjusted logistic regression models were used to measure the associations between patterns and RA.

**Results:**

Three major dietary patterns were identified. High‐level adherence to Western pattern had a positive association with RA (multivariable‐adjusted OR tertile 3 vs. 1:1.95; 95% CI: 1.09–3.92; *p*‐trend: .046), while the healthy pattern was inversely related to RA (multivariable‐adjusted OR tertile 3 vs. 1:0.12; 95% CI: 0.03–0.44; *p*‐trend: .001). No significant association was observed between the traditional pattern and RA.

**Conclusions:**

Our findings revealed that people with dietary behaviors close to the Western dietary pattern are more likely to develop the disease. However, adhering to healthy and well‐balanced dietary patterns rich in whole grains, low‐fat dairies, white meats, eggs, fruits, vegetables, tea, and vegetable oils was found to be inversely correlated with the risk of RA.

## Graphical abstract

Considering the prominent role of the overall diet in rheumatoid arthritis pathogenesis, the current study aimed to investigate the relationship between three major dietary patterns (Western dietary pattern, healthy dietary pattern, and traditional dietary pattern) and the risk of rheumatoid arthritis in newly diagnosed patients.

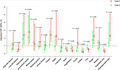

## 1 INTRODUCTION

Assessing the role of nutrients and dietary components in the development of chronic diseases is an age‐old endeavor (Calder et al., 2011; Mahalle et al., 2016; Vahid et al., 2015). However, “we don't eat nutrients, we eat foods,” and in fact, we eat foods in certain patterns, which sufficiently illustrates why the single nutrient or food approach is no longer useful in the face of chronic diet‐related diseases (Jacques & Tucker, 2001; Schulze et al., 2018). Taking a closer look, due to the highly interrelated nature of food exposures, it is almost impossible to separately evaluate the effects of nutrients and food ingredients on health and disease (Hu, 2002). Therefore, these limitations have radically shifted the primary focus of recent dietary guidelines from these traditional approaches to everything we eat and drink—dietary patterns as a whole (U.S. Department of Health and Human Services, & U.S. Department of Agriculture, 2015). A healthy dietary pattern, considering the overall individuals' diet, supports nutrient adequacy, reduces the risk of chronic diet‐related diseases, and promotes health (Kant, 2004; Millen et al., 2005; Okubo et al., 2010).

Rheumatoid arthritis (RA) is a chronic inflammatory autoimmune disorder that can eventually result in painful joint swelling, bone erosion, and early onset of significant body function impairments (Szekanecz et al., 2016). RA is afflicted approximately 1% of the world population and 37% of Iranians (Davatchi et al., 2016; Sweeney & Firestein, 2004). Moreover, about 3 out of every 10,000 people are annually diagnosed as new RA cases (Spector, 1990). Evidently, diet stands out as a potential environmental exposure associated with the RA risk (He et al., 2016; Skoczyńska & Świerkot, 2018; Sundström et al., 2015). Previous studies provided some insights into the link between dietary patterns and RA. Western dietary patterns, for example, rich in sweet snacks, high‐fat meats, and meat by‐products, refined grains, and high‐fat dairies, have been found to be positively related to the risk of developing RA (Rezazadeh et al., 2019). Inversely, some prior research suggests that people following healthy diets such as Mediterranean diets were less likely to develop the disease (Johansson et al., 2018). While some other studies present conflicting results and provide no link between the Mediterranean diet and RA (Hu et al., 2015), however, data regarding the correlation between food patterns and RA are still limited and conflicting and need further investigations. Furthermore, no attempt has yet been made to assess the traditional Iranian diet in RA patients. Thus, considering the prominent role of the overall diet in RA pathogenesis, the current study aimed to investigate the relationship between three major dietary patterns and the risk of RA in newly diagnosed patients.

## 2 MATERIALS AND METHODS

### 2.1 Study design and participants

The current case–control study conducted on 50 patients with RA attended the Rheumatology Clinic in Mashhad, Iran, and 100 healthy individuals from February 2017 to September 2018. The cases consisted of Iranian adults with RA who had newly been diagnosed by a rheumatologist and were selected through a simple random sampling technique. The controls were selected from adults with no joint or connective tissue diseases, including RA. Cases and controls were frequency‐matched on age (±5 years) and sex. The current study was implemented in accordance with the Helsinki declaration after approving by the local Ethics Committee at Mashhad University of Medical Sciences, Mashhad, Iran (ID: IR.MUMS.MEDICAL.REC.1397.327). All participants were provided with verbal and written explanations of the study objectives and methodology, and then, the informed consent was obtained from them.

### 2.2 Inclusion and exclusion criteria

The inclusion criteria for cases were as follows: adult patients aged ≥18 years; clinical diagnosis of RA by a rheumatologist using the revised criteria for RA classification presented by the American Rheumatism Association in 1987; absence of any connective tissue or joint disease, except RA; the recent RA clinical diagnosis (<6 months); and willingness to cooperate in the study. The inclusion criteria for controls were as follows: adult aged ≥18 years; absence of any connective tissue or joint disease; and willingness to cooperate in the study. The exclusion criteria were as follows: any certain conditions (e.g., taking certain medicines except anti‐inflammatory drugs or alcohol consumption) that might affect the individuals' nutritional status or any disease (e.g., severe cardiovascular diseases such as stroke and myocardial infarction, severe endocrine disorders such as diabetes) that can affect dietary intake or other dietary factors considerably; and following certain diets, such as weight gain diet, very‐low‐calorie diet or ketogenic diet, vegetarian diet during the year prior to the interview, to avoid heterogeneity in dietary intake within groups, thus decreasing overall “noise” in relation to the dietary “signal” (i.e., the true difference associated with diet in relation to RA.

### 2.3 Dietary intake assessment

The individuals' usual food intake during the last year was evaluated by completion of a valid 168‐item semi‐quantitative food frequency questionnaire (FFQ) through an in‐person interview (Mirmiran et al., 2010). Prior study showed the validity and reliability of this questionnaire for the extraction of food groups among the Iranian adult population (Esfahani et al., 2010). Participants were asked to report the consumption frequency of each food item (daily, weekly, monthly, or annually) according to its standard unit or portion size in the questionnaire. Afterward, the dietary data generated from FFQ were analyzed using Nutritionist IV software (First Databank Inc., Hearst Corp.) and the average daily intake of total energy, nutrients, and food groups was estimated. Then, individuals who reported the daily energy intake of <800 Kcal or >4,200 Kcal or reported not having consumed more than 40% of the food items in the semi‐quantitative FFQ in the year before the interview were excluded from the final analysis. To extract major dietary patterns using factor analysis, one hundred and sixty‐eight food items from FFQ were grouped into nineteen food groups based on primary ingredients or culinary usage (Soleimani et al., 2019).

### 2.4 Physical activity assessment

Giving the significant impact of physical activity on health, validated self‐report International Physical Activity Questionnaire‐Short Form (IPAQ‐SF) has been applied to assess the physical activity habits of individuals (Moghaddam et al., 2012). This questionnaire consists of 4 generic items to obtain the duration and frequency of individuals' physical activity in a continuum of intensity levels between sedentary and vigorous over the past 7 days. Total physical activity was then estimated in metabolic equivalent hours per week (MET‐h/week).

### 2.5 Anthropometric measurements

The participants' weight was measured in light clothing and no shoes using a digital scale (Seca 831) with an accuracy of 100 g. The height was measured in a standing position, without shoes using a wall‐mount measuring tape with an accuracy of 0.5 cm. Body mass index (BMI) was then calculated by dividing the weight (in kilograms) by the height (in meters) squared. Finally, we measured waist circumference (WC) at the minimum circumference between the iliac crest and the rib cage.

### 2.6 Statistical analysis

All statistical analyses were performed with the use of SPSS software, version 16. Kolmogorov–Smirnov test was used to determine the normality of quantitative variables. Data were presented as means and standard deviations for quantitative variables with normal distribution, as medians and interquartile ranges for quantitative variables with skewed distribution, or as frequencies for qualitative variables.

Factor analysis was used to identify dietary patterns from 19 main food groups. We required at least 54 eligible participants for the dietary pattern extraction using factor analysis, according to previous recommendations in which at least three participants are needed for each variable (Williams et al., 2010). We used the Kaiser–Meyer–Olkin (KMO) measure of sampling adequacy and Bartlett's test of sphericity to test the suitability of the intercorrelation among main food groups for factor analysis. The number of retained dietary patterns was determined according to the eigenvalue greater than one rule and scree plot of eigenvalue. Then, the factor loading matrix was rotated with the use of the varimax rotation test in order to simplify the interpretation of dietary patterns. The score of dietary patterns was computed by summing up intakes of all food groups weighted by their rotated factor loadings.

Differences among tertiles of each dietary pattern were ascertained by means of a chi‐square test and an ANOVA test, followed by Tukey's test as post hoc pairwise comparison. The odds ratio for the presence of arthritis rheumatoid disease according to tertiles of each dietary pattern was determined using a binary logistic regression. We also defined three models to compute multivariable‐adjusted odds ratio as follows: Model 1: adjustment for sex, age, BMI, smoking, sleep duration, dietary supplement use, and family history of arthritis; Model 2: adjustment for physical activity and energy intake; and Model 3: all foreside covariates. A P‐value less than 0.05 was considered statistically significant.

## 3 RESULTS

From January to February 2018, 132 women and 18 men participated in the current study. The mean (±*SD*) age of participants was 41.7 ± 10.5 years (range: 19–68 years). Participants in the case group had higher weight, BMI, and WC and tended to be inactive and smoker than participants in the control group.

Factor analysis identified three distinct dietary patterns, representing 40.14% of total variance with the Kaiser–Mayer–Olkin criterion of 0.669 and a significant level of Bartlett's sphericity (*p*‐value: .001). The first dietary pattern that emphasizes refined grains, red meats, processed meats, potato, nuts, coffee, sweets, vegetable oils, and hydrogenated fats was labeled Western pattern. The second dietary pattern that emphasizes whole grains, low‐fat dairies, white meats, eggs, fruits, vegetables, tea, and vegetable oils was labeled healthy pattern. The third dietary pattern that emphasizes refined grains, whole grains, high‐fat dairies, potato, legumes, nuts, vegetables, tea, sugar, and hydrogenated fats was labeled traditional pattern (Table 1).

**TABLE 1:** Factor loading matrix for major dietary patterns identified in study participants

Food groups	Western dietary pattern	Healthy dietary pattern	Traditional dietary pattern
Refined grains	0.257		0.335
Whole grains		0.534	0.328
High‐fat dairies			0.679
Low‐fat dairies		0.275	
Red meats	0.827		
Processed meats	0.929		
White meat		0.460	
Potato	0.591		0.274
Eggs		0.540	
Legumes			0.584
Nuts	0.335		0.341
Fruits		0.256	
Vegetables		0.678	0.318
Coffee	0.888		
Tea		0.509	0.542
Sugars			0.267
Sweets	0.746		
Vegetable oils	0.476	0.525	
Hydrogenated fats	0.369		0.625
Variance explained	20.97%	10.27%	9.17%

Note

Absolute values <0.2 are not displayed for simplicity.

Table 2 illustrates the participants’ characteristics according to tertiles of each dietary pattern. In Western pattern, participants in the highest tertile had a significantly higher BMI and WC than those in the other two tertiles. But, BMI and WC were significantly lower among participants in the highest tertiles of healthy pattern than those in the other two tertiles.

**TABLE 2:** General characteristics of study participants across tertiles (*T*) of dietary pattern scores

Variables	Western dietary pattern score			*p*	Healthy dietary pattern score			*p*	Traditional dietary pattern score			*p*
	*T*_1_ (lowest) (*n* = 50)	*T*_2_ (*n* = 50)	*T*_3_ (highest) (*n* = 50)		*T*_1_ (lowest) (*n* = 50)	*T*_2_ (*n* = 50)	*T*_3_ (highest) (*n* = 50)		*T*_1_ (lowest) (*n* = 50)	*T*_2_ (*n* = 50)	*T*_3_ (highest) (*n* = 50)	
Female; *n* (%)	43 (86)	45 (90)	45 (90)	.93	43 (86)	44 (88)	46 (92)	.91	44 (88)	47 (94)	42 (84)	.17
Age; years	44.4 ± 10.9	37.7 ± 9.4*	43.1 ± 10.2^&^	.003	44.1 ± 10.6	38.8 ± 10.6*	42.2 ± 9.8^&^	.035	44.5 ± 11	38.8 ± 9.5*	41.7 ± 10.5^&^	.026
Weight; Kg	64.13 ± 9.98	63.12 ± 8.39	64.35 ± 9.85	.78	62.09 ± 8.29	63.22 ± 8.74	66.21 ± 10.62	.075	64.13 ± 9.98	63.13 ± 8.39	64.35 ± 9.85	.78
BMI; kg/m^2^	23.9 ± 2.9	25.6 ± 2.9*	26.9 ± 2.6^*&^	.012	27.2 ± 3.6	25.5 ± 2.8*	23.9 ± 2.8^*&^	.006	25.1 ± 3.5	24.7 ± 3.1	24.1 ± 2.9	.26
Waist circumference; cm	83.7 ± 5.5	86.9 ± 6.6*	88.2 ± 6.6^*&^	.039	88.6 ± 8	86.3 ± 8.2*	83.1 ± 11.9^*&^	.031	87.3 ± 8.2	83.5 ± 6.6	88.1 ± 12.5	.055
Physical activity; MET‐hr/week	7.26 ± 3.59	5.51 ± 2.74	6.17 ± 4.59	.55	5.15 ± 3.75	6.06 ± 2.48	7.76 ± 4.53	.09	5.9 ± 3.84	5.87 ± 3.25	6.17 ± 4.07	.08
Sleep duration; hr/day	7.18 ± 1.56	7.15 ± 1.27	7.38 ± 1.67	.70	6.71 ± 1.25	7.16 ± 1.11	7.75 ± 1.84^*&^	.003	7.23 ± 1.62	7.52 ± 1.55	6.96 ± 1.31	.17
Time spent sitting; hr/day	5.21 ± 1.77	5.16 ± 2.03	5.58 ± 2.44	.53	6.06 ± 2.35	5 ± 1.79	4.9 ± 1.93^*&^	.007	5.55 ± 2.42	5.56 ± 1.98	4.86 ± 1.83	.16
Smoker; *n* (%)	9 (18)	7 (14)	16 (32)	.08	8 (16)	8(16)	16 (32)	.09	13 (26)	12 (24)	7 (14)	.25
Current supplement use; *n* (%)	23(46)	20(40)	22 (44)	.78	20(40)	24(48)	21(42)	.71	16 (32)	28 (56)	20 (40)	.056
Family history of RA; *n* (%)	11 (22)	11 (22)	18 (36)	.23	12 (24)	11(22)	17(34)	.40	14 (28)	11(22)	15(30)	.66
University education; *n* (%)	17 (34)	11 (22)	19 (38)	.21	16 (32)	16 (32)	15 (30)	.93	17 (34)	13 (26)	17 (34)	.60

Abbreviaions: BMI, body mass index; MET, metabolic equivalents; RA, rheumatoid arthritis.

Data are presented as means ± *SD* or frequencies. *p* values were obtained from ANOVA with Tukey's test as post hoc pairwise comparison for quantitative variables and chi‐square test for qualitative variables.

* *p* < .05, significant difference from the first tertile (*T*_1_)

^&^ *p* < .05, significant difference from the second tertile (*T*_2_).

Table 3 illustrates odds ratio for the presence of arthritis rheumatoid disease (AR) according to tertiles of each dietary pattern. Participants in the highest tertiles of Western pattern had 2.5 points greater likelihood of having AR than those in the lowest tertiles (*p*‐value: .001). A significant upward trend in the odds of AR was found across tertiles of Western pattern (*p*‐trend = .005). Although the positive association between Western pattern and having AR was attenuated by adjusting potential confounding factors, it was still statistically significant (*p*‐trend: .046). In healthy pattern, participants in the highest tertile had about 70% lower likelihood of having AR than those in the lowest tertile (*p*‐value: .001). There was a significant inverse trend in the odds of AR across tertiles of healthy pattern. The negative association between healthy pattern and having AR remained statistically significant when the odds ratio was adjusted for potential confounding factors (*p*‐trend: .001). Unlike Western and healthy dietary patterns, no significant association was found between traditional dietary pattern and having AR in both crude and adjusted models.

**TABLE 3:** Characteristics of dietary intakes across tertiles (*T*) of dietary pattern scores

Variables	Western dietary pattern score			*p*	Healthy dietary pattern score			*p*	Traditional dietary pattern score			*p*
	*T*_1_ (lowest) (*n* = 50)	*T*_2_ (*n* = 50)	*T*_3_ (highest) (*n* = 50)		*T*_1_ (lowest) (*n* = 50)	*T*_2_ (*n* = 50)	*T*_3_ (highest) (*n* = 50)		*T*_1_ (lowest) (*n* = 50)	*T*_2_ (*n* = 50)	*T*_3_ (highest) (*n* = 50)	
Total energy intake; Kcal/day	1973 ± 579	2,207 ± 371*	2,485 ± 520^*&^	.001	2079 ± 579	2,236 ± 392	2,348 ± 575	.47	1930 ± 532	2,265 ± 363*	2,469 ± 556^*&^	.001
Carbohydrate; % of energy	56.79 ± 7.96	54.24 ± 6.20*	51.46 ± 4.94^*&^	.001	54.09 ± 7.16	53.34 ± 5.09	54.93 ± 7.82	.51	51.92 ± 8.07	54.50 ± 5.74*	56.1 ± 6.13^*&^	.008
Fat; % of energy	28.5 ± 6.3	30.9 ± 4.79*	35.13 ± 4.87^*&^	.001	31.16 ± 6.3	32.08 ± 4.67	31.36 ± 6.85	.72	32.41 ± 4.68	30.87 ± 5.30	31.1 ± 7.65	.42
Protein; % of energy	14.71 ± 3.62	14.89 ± 2.700	13.41 ± 2.09	.16	14.74 ± 3.35	14.57 ± 2.46	13.7 ± 2.85	.16	15.67 ± 3.24	14.61 ± 3.1*	12.67 ± 2.9^*&^	.004
Dietary fiber; gr/1,000 Kcal	18.76 ± 3.34	16.35 ± 2.42*	15.89 ± 2.38^*&^	.001	17.42 ± 2.74	17.55 ± 2.60	16.97 ± 3.82	.62	15.2 ± 2.29	17.32 ± 2.04*	18.9 ± 4.24^*&^	.005
SFAs; gr/1,000 Kcal	13.97 ± 3.75	15.63 ± 3.57*	17.65 ± 3.59^*&^	.001	18.03 ± 4.1	15.45 ± 2.96*	14.7 ± 3.45^*&^	.001	17.02 ± 4.74	15.44 ± 3.31	15.86 ± 2.59	.10
MUFAs; gr/1,000 Kcal	11.2 ± 4.41	12.98 ± 3.4*	15.61 ± 5.12^*&^	.001	11.77 ± 4.11	12.78 ± 4.82	12.46 ± 4.62	.46	12.63 ± 4.66	12.97 ± 4.93	11.78 ± 4.26	.42
PUFAs; gr/1,000 Kcal	9.32 ± 3.3	9.78 ± 2.28	9.99 ± 3.23	.059	10.42 ± 4.02	9.75 ± 2.56	9.83 ± 2.99	.053	10.22 ± 4.15	9.94 ± 2.84	9.84 ± 2.56	.83
Trans FAs; gr/1,000 Kcal	1.19 ± 0.83	0.95 ± 0.68	0.96 ± 0.63	.078	1.27 ± 1.12	0.98 ± 0.46*	0.73 ± 0.62^*&^	.001	0.71 ± 0.4	0.99 ± 0.38*	1.37 ± 0.57^*&^	.003
Cholesterol; mg/1,000 Kcal	184 ± 67.9	188 ± 46.8	191 ± 76.5	.85	188 ± 67.1	181 ± 36.9	195 ± 81.8	.54	181 ± 61.2	187 ± 71.7	196 ± 60.8	.51
Sodium; mg/1,000 Kcal	1,890 ± 551	1940 ± 512	1807 ± 556	.46	1997 ± 520	1891 ± 577	1752 ± 502	.075	2,138 ± 682	1843 ± 396*	1663 ± 412^*&^	.001
E‐DII score	−0.71 ± 0.31	−0.55 ± 0.44*	−0.26 ± 0.50^*&^	.001	−0.28 ± 0.45	−0.50 ± 0.47*	−0.74 ± 0.4^*&^	.005	−0.49 ± 0.52	−0.54 ± 0.45	−0.48 ± 0.49	.34

Abbreviations: E‐DII, energy‐adjusted dietary inflammatory index; FAs, fatty acids; MUFAs, monounsaturated fatty acids; PUFAs, polyunsaturated fatty acids; SFAs, saturated fatty acids.

Data are presented as means ± *SD*. *p* values were obtained from ANOVA with Tukey's test as post hoc pairwise comparison.

* *p* < .05, significant difference from the first tertile (*T*_1_).

^&^ *p* < .05, significant difference from the second tertile (*T*_2_).

In Western pattern, participants in the highest tertiles consumed higher quantities of energy, SFAs, and MUFAs but lower quantities of dietary fiber and had higher scores of E‐DII than those in the other two tertiles. In healthy pattern, participants in the highest tertiles consumed lower quantities of SFAs and TFAs and had lower scores of E‐DII than those in the two other tertiles. In traditional pattern, participants in the highest tertiles consumed higher quantities of energy, carbohydrates, TFAs, and dietary fiber but lower quantities of protein than those in the two tertiles (Table 4).

**TABLE 4:** Odds ratios (95% CI)^a^ for having arthritis rheumatoid disease across tertiles (*T*) of dietary pattern scores

	Western dietary pattern score			*p*	Healthy dietary pattern score			*p*	Traditional dietary pattern score			*p*
	*T*_1_ (lowest)	*T*_2_	*T*_3_ (highest)		*T*_1_ (lowest)	*T*_2_	*T*_3_ (highest)		*T*_1_ (lowest)	*T*_2_	*T*_3_ (highest)	
Crude	1	1.14 (0.76 –2.58)	2.51 (1.39 – 5.07)	.005	1	0.51 (0.23 – 0.91)	0.33 (0.14 – 0.76)	.001	1	2.98 (1.31 – 6.81)	0.31 (0.11 – 0.88)	.22
Model 1	1	1.03 (0.64 – 2.55)	2.35 (1.28 – 4.71)	.016	1	0.43 (0.19 –0.81)	0.31 (0.12 – 0.77)	.001	1	2.34 (1.03 – 5.39)	0.26 (0.08 – 0.78)	.16
Model 2	1	1.11 (0.71 – 2.79)	2.93 (1.39 – 6.09)	.006	1	0.54 (0.22 – 1.04)	0.35 (0.18 –0.98)	.019	1	3.85 (1.35 – 11.1)	0.57 (0.17 – 1.86)	.69
Model 3	1	1.13 (0.75 – 2.65)	3.21 (1.41 – 5.25)	.007	1	0.49 (0.21 – 0.81)	0.32 (0.13 – 0.74)	.008	1	2.9 (1.27 – 6.6)	0.31 (0.11 – 0.86)	.82
Model 4	1	1.08 (0.73 – 2.41)	2.27 (1.39 – 4.66)	.002	1	0.51 (0.23 – 0.84)	0.31 (0.12 – 0.73)	.007	1	2.88 (1.24 – 6.67)	0.31 (0.11 – 0.89)	.71
Model 5	1	0.98 (0.49 – 2.47)	2.04 (1.18 – 4.16)	.031	1	0.38 (0.17 – 0.68)	0.25 (0.15 – 0.53)	.001	1	2.45 (0.96 – 6.25)	0.27 (0.09 – 0.81)	.92
Model 6	1	0.91 (0.43 – 2.35)	1.92 (1.09 – 3.92)	.046	1	0.28 (0.14 – 0.65)	0.12 (0.03 – 0.44)	.001	1	1.74 (0.52 – 6.1)	0.31 (0.13 – 1.21)	.69

Note

Model 1: Adjusted for sex, age, and BMI.

Model 2: Adjusted for sleep duration, time spent sitting, physical activity, and smoking.

Model 3: Adjusted for dietary supplement use.

Model 4: Adjusted for family history of arthritis.

Model 5: Adjusted for energy intake.

Model 6: Adjusted for the abovementioned variables

^a^ Odds ratios (95% confidence interval) were obtained using binary logistic regression.

**FIGURE 1:**
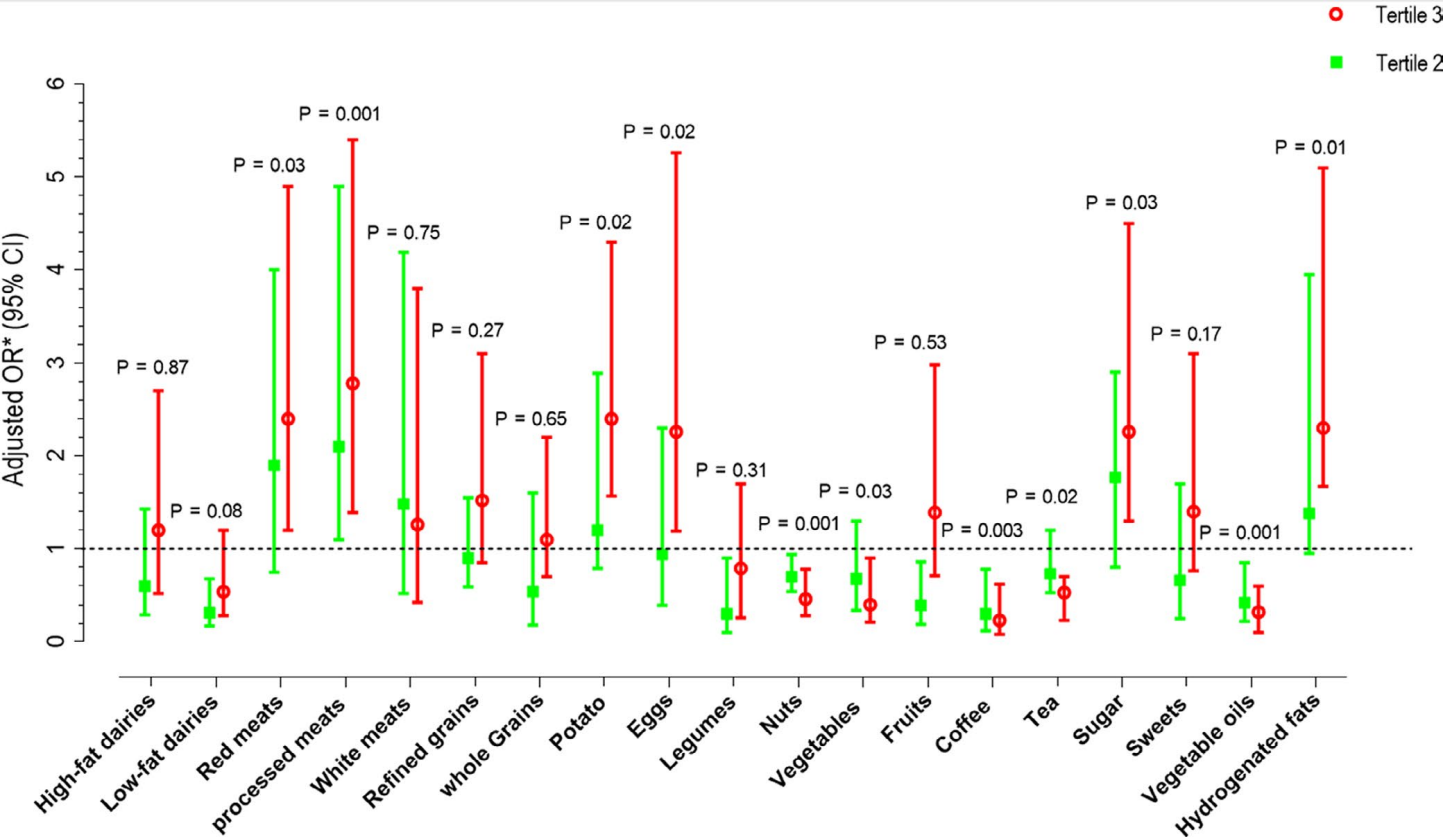
Multivariable‐adjusted odds ratio for arthritis rheumatoid diseases across tertiles of main food groups * Adjusted for sex, age, BMI, smoking, physical activity, family history, dietary supplements use, and energy intake Kaiser–Meyer–Olkin Measure of sampling adequacy = 0.66

The odds ratio for the presence of AR according to tertiles of food groups making up the dietary patterns is shown in Figure 1. Participants in the highest tertile of intake of red meats, processed meats, potato, eggs, sugar, and hydrogenated fats had significantly higher odds of having AR than those in the lowest tertile of these food groups. A significant upward trend in the odds of AR was found across tertiles of red meats (*p*‐trend: .03), processed meats (*p*‐trend: .001), potato (*p*‐trend: .02), eggs (*p*‐trend: .02), sugar (*p*‐trend: .03), and hydrogenated fats (*p*‐trend: .01). On the other hand, participants in the highest tertile of intake of nuts, vegetables, coffee, tea, and vegetable oils had significantly lower odds of having AR than those in the lowest tertile of these food groups. A significant inverse trend was observed in the odds of AR across tertiles of nuts (*p*‐trend: .001), vegetables (*p*‐trend: .03), coffee (*p*‐trend: .003), tea (*p*‐trend: .02), and vegetable oils (*p*‐trend: .001).

## 4 DISCUSSION

In the present cross‐sectional study, three major dietary patterns were extracted: Western dietary pattern, healthy dietary pattern, and traditional dietary pattern. The overall results indicated that RA was positively correlated with the Western diet and negatively correlated with the healthy diet, both independent of potentially confounding factors. However, no significant correlation was found between the traditional dietary pattern and the risk of RA. To best of our knowledge, there are still limited data regarding the correlation between dietary patterns and RA risk. However, an extensive literature was developed on the relationship between food components and RA. Our findings revealed that people with dietary behaviors close to the Western dietary pattern rich in refined grains, red and processed meats, sweets, and hydrogenated fats are more likely to develop the disease. This association may be due to the potential inflammatory effects of food ingredients on the disease. Indeed, certain components of the Western diet, such as refined grains and sweets, are high in glycemic load, resulting in abnormal lipid profiles, overproduction of free radicals, insulin resistance, and metabolic syndrome. These abnormalities may ultimately lead to extensive systemic inflammation and chronic inflammatory diseases (Chung et al., 2008; Hu et al., 2015; Liu et al., 2002). In parallel to this finding, a study of 1,209 American adults aged 20–30 found that consuming sugary drinks rich in fructose such as sweetened soft drinks and fruit drinks at least five times a week increased the risk of arthritis by three times independent of potentially confounding factors including the level of plasma glucose, other dietary factors, smoking, or physical activity (DeChristopher et al., 2016). Besides, the high consumption of red meat has been suggested as a major and independent risk factor in the expression of RA. One possible explanation lies in the activation of inflammatory pathways and the excess generation of free radicals due to certain meat agents such as fat and nitrite (Grant, 2000). Furthermore, excess iron intake following high meat consumption increases the synovial involvement, which can partly explain the role of meat in the etiology of arthritis (Morris et al., 1986). Analysis of data from the European Prospective Investigation of Cancer in Norfolk [EPIC‐Norfolk] revealed that higher consumption of red meats, meat by‐products, and total proteins was positively correlated with the risk of inflammatory polyarthritis (Pattison et al., 2004). Diets high in saturated fatty acids (SFAs) mostly derived from animal products can also trigger inflammatory diseases by activating toll‐like receptors and initiating inflammatory cascades (Rocha et al., 2016). Previous literature has found that the greater the reduction in fatty acid consumption, the higher the anti‐inflammatory adipokines, and the lower the pro‐inflammatory adipokines tend to be (Vannice & Rasmussen, 2014). A healthy dietary pattern was found in our results to be inversely correlated with the risk of RA. As an essential part of a healthy and well‐balanced dietary pattern, whole grains, vegetables, fruits, and fish are packed with a variety of antioxidants and micronutrients that have potential in modulating inflammatory responses in RA. In the prospective population‐based study of EPIC‐Norfolk by Pattison et al. (2004), it was revealed that diets rich in fruits and vegetables, particularly varieties rich in vitamin C, were strongly linked to a three times increase in inflammatory arthritis. Furthermore, regular consumption of mushrooms and citrus fruits has been found to have protective effects against the development of RA (He et al., 2016). Besides, in the case–control study conducted in Greece, cooked vegetable and olive oil were found to be inversely and independently correlated with RA risk (Linos et al., 1999). In 2016, Aparicio‐Soto et al. described extra virgin olive oil, “a key functional food for prevention of immune‐inflammatory diseases.” This protective property of olive oil is significantly explained by the immune‐modulatory effect of its main contents such as phenolic compounds and monounsaturated fatty acid (MUFA), particularly oleic acid (Aparicio‐Soto et al., 2016). Our results also revealed that adhering to dietary behaviors close to the traditional dietary pattern rich in refined grains, whole grains, high‐fat dairies, potato, legumes, nuts, vegetables, tea, sugar, and hydrogenated fats has no correlation with the RA risk. To a large extent, this finding can be explained by the complex nature of the traditional diet, which includes both healthy and unhealthy foods. The limitation of the current study is that it was not able to evaluate the causality relationship between dietary patterns and RA due to its observational design in which both exposure and outcome are assessed at the same time point. However, our study was strengthened with well‐matched control groups in terms of demographic variables and with adjustment for potential confounding factors.

## 5 CONCLUSION

Taken collectively, our findings revealed that people with dietary behaviors close to the Western dietary pattern loaded with refined grains, red and processed meats, sweets, and hydrogenated fats are more likely to develop the disease. However, adhering to healthy and well‐balanced dietary pattern rich in whole grains, low‐fat dairies, white meats, eggs, fruits, vegetables, tea, and vegetable oils was found to be inversely correlated with the risk of RA. However, no significant association was found between the traditional dietary pattern and the risk of RA. These associations should be tested again in future studies with a longitudinal design.

## CONFLICT OF INTEREST

The authors declare no conflict of interest.

## TRANSPARENCY DECLARATION

The lead author affirms that this manuscript is an honest, accurate, and transparent account of the study being reported. The reporting of this work is compliant with STROBE2 guideline. The lead author affirms that no important aspects of the study have been omitted and that any discrepancies from the study as planned have been explained.
